# Targeting STING: From antiviral immunity to treat osteoporosis

**DOI:** 10.3389/fimmu.2022.1095577

**Published:** 2023-01-18

**Authors:** Zhonghua Gao, Zhongguo Gao, Hao Zhang, Shoubo Hou, Yunhua Zhou, Xiangjie Liu

**Affiliations:** ^1^Department of Geriatrics, Liyuan Hospital, Tongji Medical College, Huazhong University of Science and Technology, Wuhan, Hubei, China; ^2^Department of Medical Laboratory Technology, School of Biomedical Engineering, Hubei University of Medicine, Shiyan, Hubei, China; ^3^Department of General Practice, General Hospital of Central Theater Command, Wuhan, Hubei, China; ^4^Department of Wound Repair Surgery, Liyuan Hospital, Tongji Medical College, Huazhong University of Science and Technology, Wuhan, Hubei, China

**Keywords:** osteoporosis, STING, IFN-β, NF-κB, type H vessels

## Abstract

The cGAS-STING signaling pathway can trigger innate immune responses by detecting dsDNA from outside or within the host. In addition, the cGAS-STING signaling pathway has emerged as a critical mediator of the inflammatory response and a new target for inflammatory diseases. STING activation leads to dimerization and translocation to the endoplasmic reticulum Golgi intermediate compartment or Golgi apparatus catalyzed by TBK1, triggers the production of IRF3 and NF-κB and translocates to the nucleus to induce a subsequent interferon response and pro-inflammatory factor production. Osteoporosis is a degenerative bone metabolic disease accompanied by chronic sterile inflammation. Activating the STING/IFN-β signaling pathway can reduce bone resorption by inhibiting osteoclast differentiation. Conversely, activation of STING/NF-κB leads to the formation of osteoporosis by increasing bone resorption and decreasing bone formation. In addition, activation of STING inhibits the generation of type H vessels with the capacity to osteogenesis, thereby inhibiting bone formation. Here, we outline the mechanism of action of STING and its downstream in osteoporosis and discuss the role of targeting STING in the treatment of osteoporosis, thus providing new ideas for the treatment of osteoporosis.

## 1 Introduction

The stimulator of interferon genes (STING, also known as MITA, MPYS, ERIS, and TMEM173) is a pattern recognition receptor (PRR) that recognizes nucleic acids of pathogenic microorganisms or cell membrane components, among others (1). It acts as the first line of defense of cells against pathogenic invasion. Initially found in the endoplasmic reticulum (ER) membrane of the innate immune cells and later also found to be expressed in T cells and other cells, it recognizes released DNA and triggers innate immune activation with essential functions in infection, inflammation, and cancer (2). STING, a necessary protein of natural immunity, plays a crucial role in antiviral immunity by activating nuclear factor-kappa B (NF-κB) and interferon regulatory factor 3 (IRF3) and producing type I interferon (IFN-I) independently of Toll-like receptors (TLRs, another type of PRR) (2).

The cGAS-STING in the innate immune response is vital in defending against pathogenic microbial invasion (3). In addition to its antiviral immune function, STING can cause inflammatory and autoimmune diseases (4). Activation of STING causes the transcription of inflammatory genes and increases pro-inflammatory cytokines. The increase of overpowering pro-inflammatory factors then causes inflammatory and autoimmune diseases (5). Therefore, STING is also the inflammatory protein that triggers chronic inflammation (6). The cGAS-STING pathway mediates the cellular inflammatory response and thus plays a crucial role in the pathogenesis of inflammatory diseases such as ischemic myocardial infarction (MI), nonalcoholic steatohepatitis (NASH), traumatic brain injury (TBI), and silicosis (7). A chronic low-grade inflammatory state accompanies aging. The cGAS-STING pathway can also induce the senescence-associated secretory phenotype (SASP) through the accumulation of cytoplasmic DNA during aging, which leads to the development of aging-related diseases (8).

**Figure 1:**
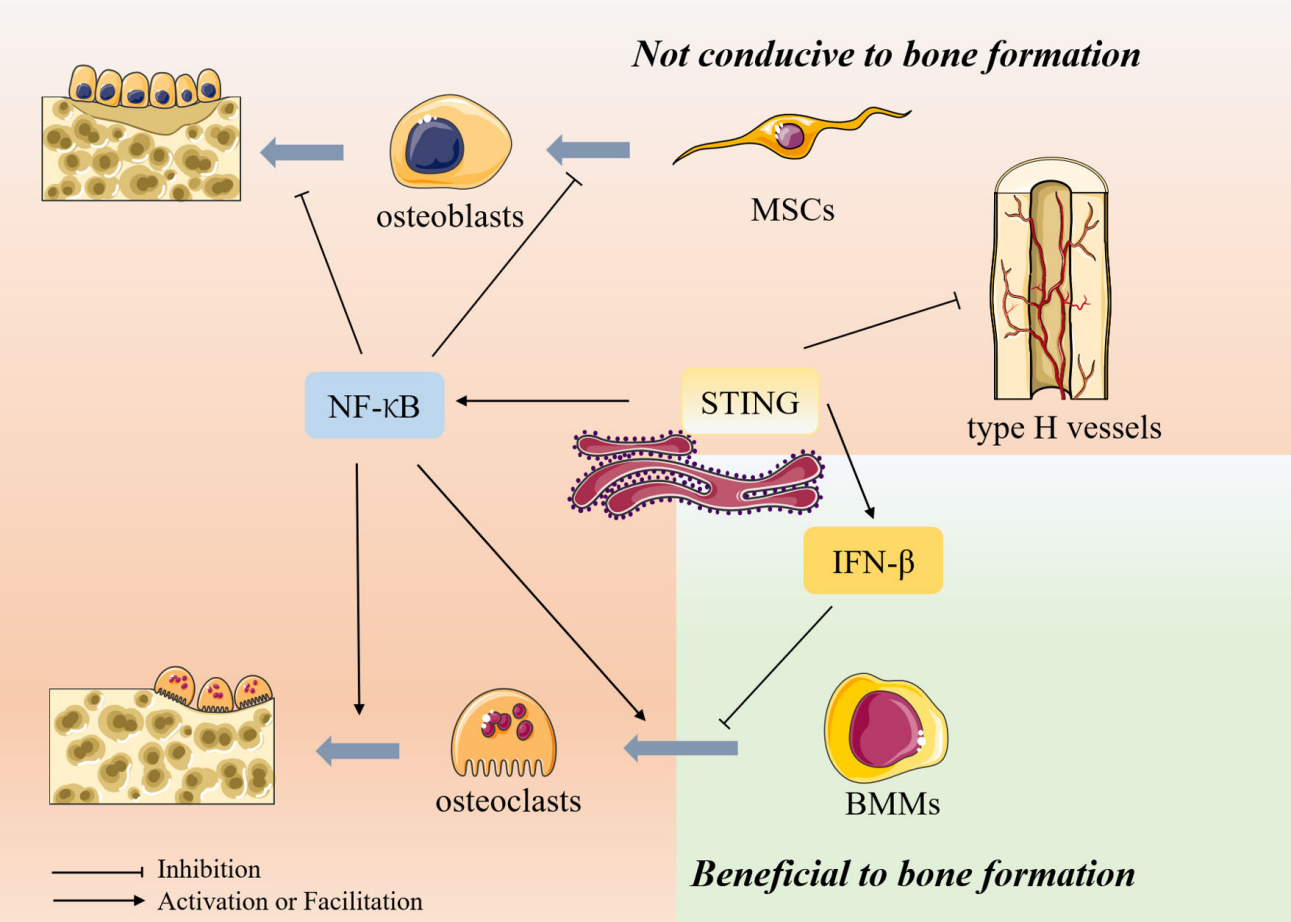
The role of STING in bone metabolism Bone metabolism is mainly composed of osteoblast-mediated bone formation and osteoclast-mediated bone resorption. In addition, type H vessels also can induce bone formation and thus participate in bone remodeling. Osteoblasts are differentiated from mesenchymal stem cells (MSCs), whereas osteoclasts are differentiated from bone marrow macrophages. STING can act as an upstream of NF-κB, stimulating its activation and transcription and thus exerting biological effects. NF-κB inhibits the differentiation of MSCs toward osteogenesis and inhibits osteoblast activity, thus inhibiting bone formation. In osteoclasts, NF-κB can promote osteoclast production and activity, thereby promoting bone resorption. IFN-β is also a downstream target of STING. However, unlike NF-κB, although IFN-β is induced in osteoclasts after activation by STING, it can inhibit osteoclast activation through its negative feedback, thereby inhibiting bone resorption. In addition, STING can inhibit the formation of type H vessels, inhibiting bone formation.

Hundreds of millions worldwide are affected by bone-related diseases such as osteoporosis, degenerative disc disease, and rheumatoid arthritis. Osteoporosis is an age-related degenerative disease of bone, mainly due to changes in the bone microenvironment and structural degeneration, resulting in reduced bone density (9). It seriously endangers patients’ quality of life and lives due to the extreme risk of fractures and others and causes a substantial financial burden on society. Osteoporosis is also a sterile inflammatory disease characterized by the activation of NF-κB at the molecular level, which promotes osteoclast-mediated bone resorption and inhibits osteoblast-induced bone formation (10). Notably, STING can act as an upstream of NF-κB, stimulating its activation and transcription, thus mediating pro-inflammatory effects and playing a role in the pathogenesis of osteoporosis **(** **Figure 1****)**. IFN-β is also a downstream target of STING. However, unlike NF-κB, although IFN-β is induced by STING activation in osteoclasts, it can inhibit osteoclast activation through negative feedback (11). In addition, STING can act on vascular endothelial cells (ECs) to regulate the formation of type H vessels, which can control bone formation. STING activation can impair their formation and thus affect bone formation (12) (**Table 1**). Therefore, the role of STING in osteoporosis deserves further investigation to determine how to target STING for osteoporosis treatment.

**Table 1:** Summary of research on targeting STING and it signaling pathways in bone metabolism.

Disease	Interventions	Mechanism &Target	Model	Effects
OA	Deficient in STING (13)	Inhibiting STING / IFN-I signaling	DNase II^-/-^/ IFNAR^-/-^ double-knockout arthritis mice	Inhibiting abnormal bone formation
	Itaconate (14)	Inhibiting STING/NF-κB axis, Promoting M2 polarization in macrophages	OA mouse	Inhibiting chondrocyte senescence and ECM degeneration, Attenuating osteoarthritis
IVDD	Lipopolysaccharide (LPS) (15)	Activating cGAS/STING pathway	vertebral inflammation-induced caudal IVDD (VI-IVDD) rat	Building a novel model of VI-IVDD
	STING knock-down (16)	Inhibiting cGAS/STING pathway	puncture-induced IVDD rat	Alleviating IVDD development
	Epigallocatechin-3-Gallate (17)	Inhibiting cGAS/STING/ NLRP3 pathway	H_2_O_2_ -Treated NP cells	Protecting NP cells from apoptosis
Bone Loss (including OP)	STING agonists (18) (DMXAA, ADU-S100)	Activating STING/IFN-I signaling	Lewis lung carcinoma or breast cancer -induced bone loss mice	Reducing bone loss
	CDNs (19)	Activating STING/IFN-β signaling	RANKL-induced BMMs, calvarial implantation mouse	Inhibiting osteoclast differentiation and bone resorption
	Tmem173(STING) overexpression (20)	Overexpressing STING	RANKL-induced BMMs	Inhibiting osteoclast differentiation
	RTA-408 (21)	Inhibiting STING /NF-κB signaling	OVX-induced bone loss	Attenuating osteoclastogenesis
Bone Fracture, Bone Defect	2',3'-cGAMP (12)	Activating STING	Bone fracture and defect mice	Inhibiting type H vessel formation, Delaying bone healing
	STING inhibitors (12) (C-176 and H-151)	Inhibiting STING		Enhancing type H vessel formation, Accelerating bone healing

## 2 cGAS-STING pathway

The cGAS-STING pathway is a significant component of the host’s innate immune response to viral infection. The cGAS senses pathogenic DNA to activate STING to modulate the type 1 interferon response to trigger a natural immune response. Herpes simplex virus 1 (HSV-1) is a double-stranded DNA virus sensed by the cGAS to activate STING and induce innate antiviral immunity (22). Similarly, other DNA viruses, such as HIV and CMV, can trigger cGAS-STING (23, 24). STING also plays a vital role in the immune response induced by RNA viruses (25). RNA viruses, like dengue virus and SARS-CoV-2, have no DNA and cannot induce cGAS autonomously. However, these RNA viruses can activate the cGAS-STING pathway by triggering intracellular mitochondrial stress damage to release mitochondrial DNA (mtDNA), thereby generating antiviral immunity (26). In addition, cGAS can sense bacterial DNA and the host’s DNA, such as senescent apoptotic cells, extracellular vesicles, and chromatin fragments (27). Thus, the cGAS-STING pathway is critical in many disease processes, including autoimmune diseases, inflammatory diseases, degenerative diseases, and cancer (5, 28).

**Figure 2:**
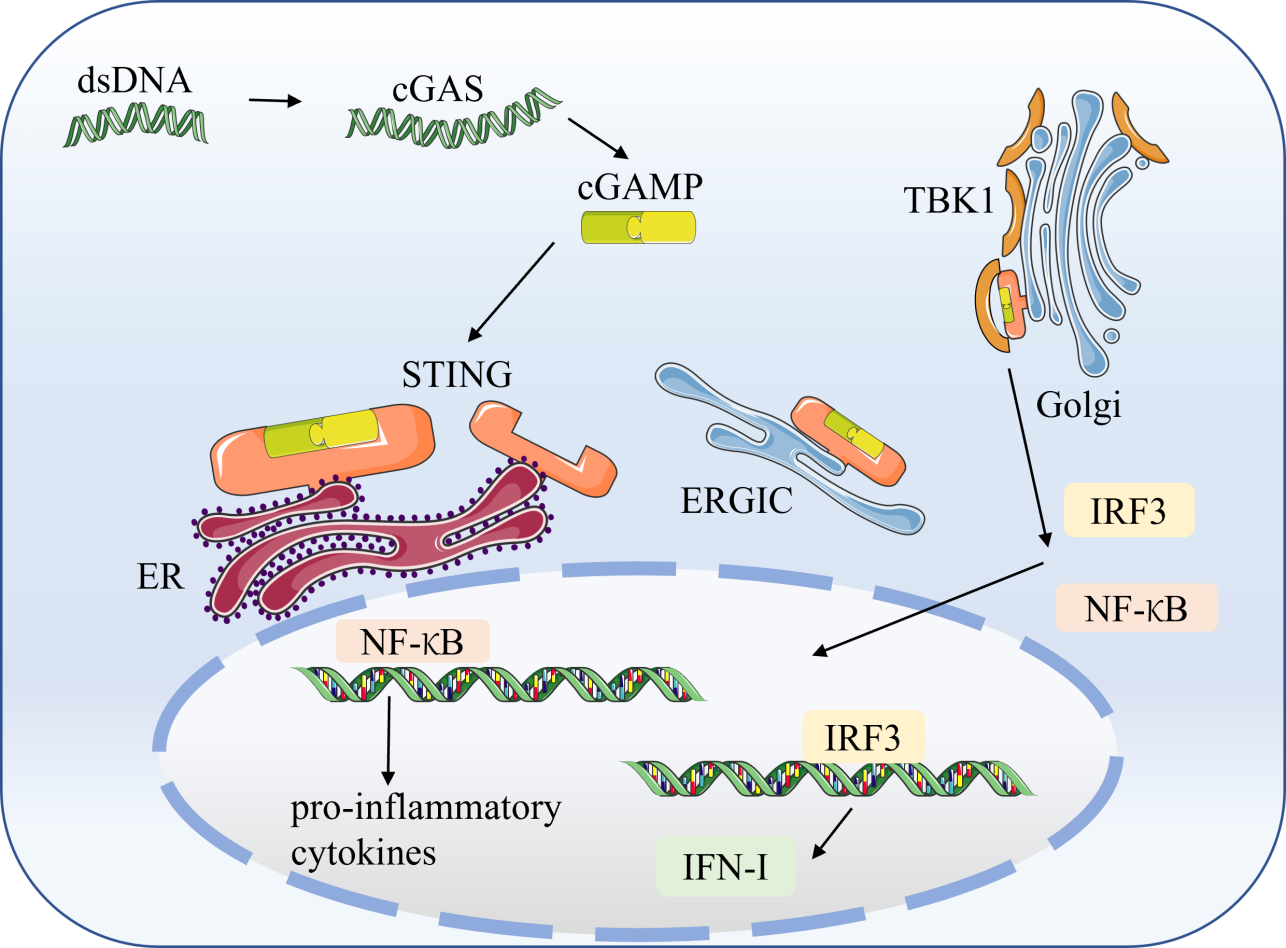
The cGAS-STING signaling pathway STING is a pattern recognition receptor (PRR) on the endoplasmic reticulum (ER) that does not bind directly to DNA. Pathogenic microorganisms and damaged host cells can release double-stranded DNA (dsDNA). The cytoplasmic DNA sensor, cyclic GMP–AMP synthase (cGAS), recognizes dsDNA and catalyzes the synthesis of cGAMP from ATP and GTP. The cGAMP binds to STING, triggering STING conformational transitions, dimerization, and translocation to the endoplasmic reticulum-Golgi intermediate compartment (ERGIC) and the Golgi apparatus (Golgi). The dimerized STING recruits TBK1, which induces the production of IRF3 and NF-κB. Subsequently, IRF3 and NF-κB translocate to the nucleus to induce the production of IFN-I and pro-inflammatory factors.

STING is a PRR on the ER that does not bind directly to DNA. Pathogenic microbes and damaged host cells can release free double-stranded DNA (dsDNA) (29). Then dsDNA is recognized by the cytoplasmic DNA sensor, the cyclic GMP-AMP synthase (cGAS) (30). The ds DNA binding to cGAS triggers the conversion of ATP and GTP to cGAMP (2′,3′-cyclic GMP–AMP) (31). The cGAMP is canonical cyclic dinucleotides (CDNs) that bind and activate STING (32). CDNs are essential second messengers produced by cyclic dinucleotide synthase, which is widely distributed and can trigger from various cellular signaling cascades, as well as being an activating ligand for STING (33, 34). The binding of cGAMP to STING triggers STING conformational transition, dimerization, and translocation to the endoplasmic reticulum-Golgi intermediate compartment (ERGIC) and Golgi apparatus (Golgi) (35). Then STING dimers recruit TBK1, which phosphorylates STING and induces IRF3 (36). STING also leads to NF-κB activation. IRF3 and NF-κB are translocated to the nucleus to induce the production of IFN-I and other cytokines involved in the host immune response (37)**(** **Figure 2****)**.

Classical STING activation induces the critical transcription factor IRF3 *via* the cGAS-STING pathway, which promotes IFN-I secretion and activates NF-κB to trigger pro-inflammatory cytokines. In recent years, atypical patterns of STING activation have also been identified. Keratinocytes generate an innate immune response within hours of etoposide-induced DNA damage, which involves the DNA sensing adapter STING but is not dependent on cGAS (38). And this non-canonical STING signaling predominantly activates NF-κB rather than IRF3, which induces IFN-I production. This also provides another way of thinking for future STING research.

Although it has been reported that cytoplasmic DNA-mediated STING-dependent inflammatory response requires activation of NF-κB *via* TBK1 (39), at the same time, activation of TBK1 can also cause IFN-β production. However, investigators have found that selective activation of NF-κB can occur in the cGAS-STING pathway, while a parallel path blocks activation of the IRF3/IFN system (40). In late 2019, SARS-CoV-2 emerged as a highly infectious coronavirus that causes a human respiratory disease called COVID-19. SARS-CoV-2 infection can cause respiratory symptoms ranging from mild to severe, resulting in lasting lung damage or death (41). One of the hallmarks of severe COVID-19 is low levels of IFN-I and high levels of expression of inflammatory cytokines or chemokines such as IL-6 and tumor necrosis factor (TNF) (42–44). This unbalanced immune response fails to limit viral transmission and leads to severe systemic symptoms. Specific activation of NF-κB and blockade of IRF3 nuclear translocation occurs in SARS-CoV-2 infected cells, and STING-targeted drugs can attenuate this NF-κB response (44). This NF-κB response is induced by mtDNA released from cellular oxidative stress injury (44, 45). MtDNA mediates the activation of cGAS-STING. Furthermore, cancer studies found that the classical NF-κB pathway in the cGAS-STING pathway enhances anti-tumor effects by promoting IFN-I expression. In contrast, the non-classical NF-κB pathway impedes anti-tumor effects by decreasing IFN-I expression (40). Activation of STING triggers NF-κB activation that can be generated independently of IFN-I.

## 3 STING/IFN-β and osteoporosis

Bone, the body’s central axis system, provides physical support and protection, is involved in calcium metabolism and endocrine regulation, and promotes the hematopoietic system in the bone marrow (10). In response to normal wear and mechanical forces as well as the aging process, bone in the adult skeleton undergoes continuous remodeling in which damaged or failing microscopic parts of the bone are removed by osteoclasts and subsequently replaced by new bone laid down by osteoblasts (46). Bone remodeling is a continuous dynamic process that includes bone formation and bone resorption activities, generally in balance, thus maintaining bone homeostasis (47). Bone homeostasis depends on the functional balance between bone-forming cells (osteoblasts) and bone-resorbing cells (osteoclasts) (48). Disruption of bone homeostasis is the frequent pathophysiological mechanism of bone metabolic diseases (49). Excessive osteoclast activity can lead to bone diseases such as osteoporosis, Paget’s disease, and rheumatoid arthritis.

**Figure 3:**
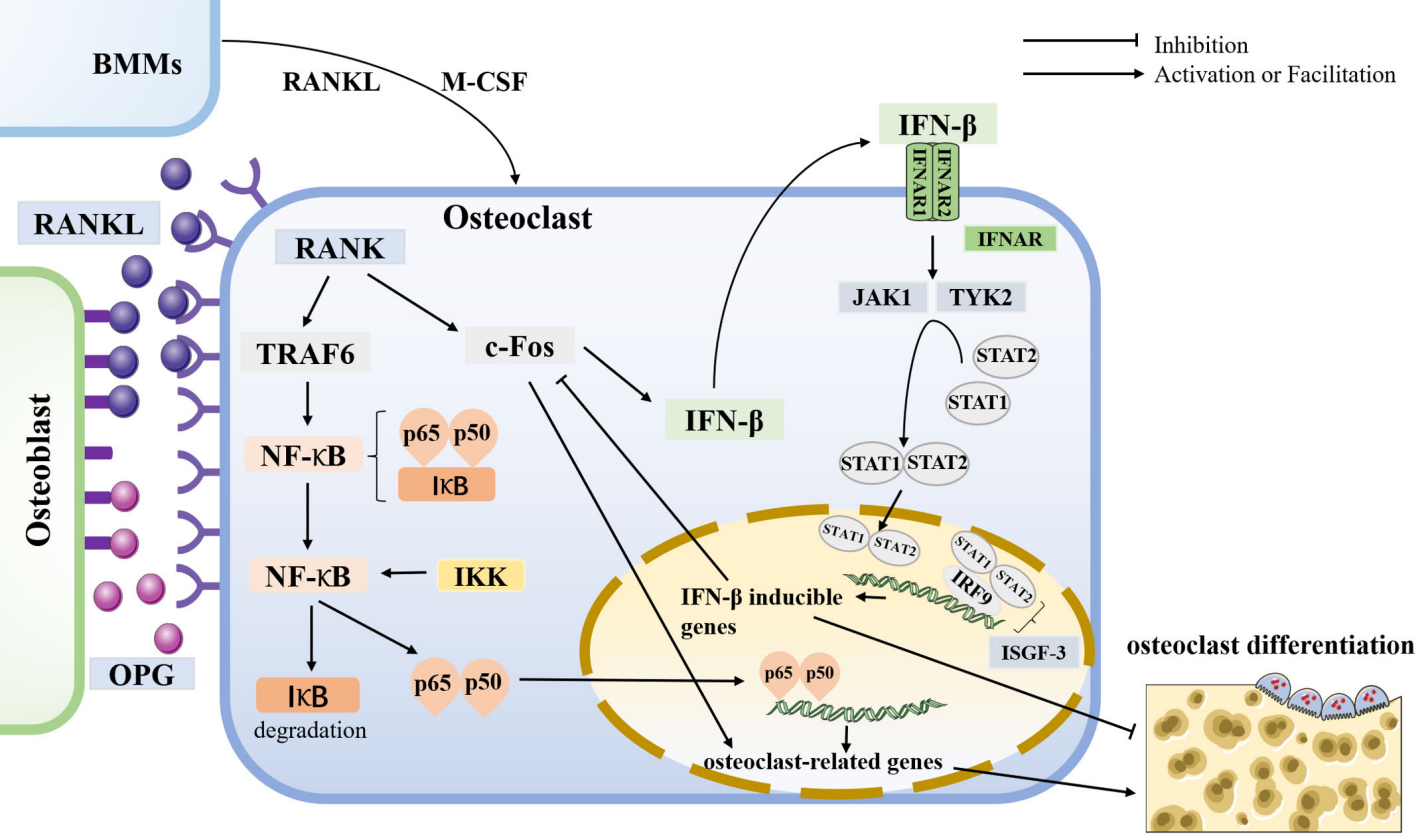
osteoclastogenesis and the role of IFN-β in it Osteoclasts are differentiated from BMMs by stimulating receptor activators for nuclear factor-κB ligand (RANKL) and macrophage colony-stimulating factor (M-CSF). Osteoblasts release RANKL and osteoprotegerin (OPG) to regulate bone homeostasis. RANK is a receptor for RANKL, expressed in osteoclasts. In osteoclasts, RANK-RANKL interaction activates downstream signaling pathways that induce the expression of osteoclast-associated genes such as c-Fos and TRAF6. In comparison, OPG binds to RANK to reduce RANK-RANKL signaling to balance bone resorption. C-Fos is essential for osteoclast differentiation. The RANK-RANKL interaction has been shown to induce IFN-β production by the c-Fos. IFN receptor (IFNAR) is a class of heterodimers on the cell membrane consisting of two subunits, IFNAR1 and IFNAR2. IFN-β binds to its receptor to activate the downstream protein kinases JAK1 and TYK2, then activating the transcription factors STAT1 and STAT2, forming a dimer that can enter the nucleus and bind to IRF9 to constitute ISGF-3, which exerts its IFN-β mediated transcriptional effects to inhibit osteoclast production. Thus, IFN-β forms negative feedback, inhibiting osteoclast differentiation. In addition, TRAF6 is essential for osteoclastogenesis and induces the production of NF-κB. In the resting state, NF-κB in the cytoplasm binds to the inhibitor protein IκB while leaving NF-κB inactive. IκB kinase (IKK) phosphorylates NF-κB to degrade IκB, and the released p65 and p50 subunits enter the nucleus for transcription of osteoclast-related genes, thus inducing bone resorption.

Osteoclast differentiation is initiated by bone marrow macrophages (BMMs) through stimulation of receptor activators of nuclear factor-κB ligand (RANKL) and macrophage colony-stimulating factor (M-CSF) (50). Osteoblasts release RANKL and osteoprotegerin (OPG) to regulate bone homeostasis. RANK, the receptor of RANKL, is expressed in osteoclasts. Furthermore, RANK-RANKL interaction activates downstream signaling pathways such as NF-κB, MAPK, and AKT, thereby inducing the expression of osteoclast-associated genes, including c-Fos and NFATc1 (51). Additional studies have shown that osteoclastogenesis generates reactive oxygen species (ROS), and these ROS can induce the activation of downstream signaling pathways, such as NF-κB and MAPK, which also play a role in osteoclast differentiation and bone resorption (52). Conversely, OPG binds to RANK to reduce RANK-RANKL signaling, thereby balancing bone resorption (53). C-Fos is essential for osteoclast differentiation, and lack of c-Fos can lead to osteosclerosis. It interacts with NFATc1, then activates multiple target genes for osteoclast function, triggering a transcriptional regulatory cascade (54). RANKL interactions have been shown to induce IFN-β production through the induction of c-Fos genes (11)**(** **Figure 3****)**.

### 3.1 Relationship between IFN-β and osteoporosis

IFN-β belongs to type I interferons. The human body produces three known types of interferons: type I, type II, and type III (55). Type I interferons are mainly IFN-α and IFN-β, secreted by innate immune cells. Type II interferons, IFN-γ, is mainly produced by activated T cells. Type III interferons include IFN-λ, whose known distribution and function are minimal. Type I interferons are mainly produced by surface or internal receptors of innate immune cells (TLRs, NLRs, RLRs, and cGAS) binding to specific antigens from outside or inside the host (56). IFN receptors (IFNAR) are a class of heterodimers located on the cell membrane and consist of two subunits, IFNAR1 and IFNAR2, and widely distributed, including monocytes, macrophages, B cells, T cells, epithelial cells, endothelial cells, and tumor cells (57). Ligand receptor binding activates downstream protein kinases JAK1 and TYK2, and kinase activation activates cytoplasmic transcription factors STAT1 and STAT2, forming a dimer that enters the nucleus to assist IRF9 in transcribing some downstream effector genes (56). Type I interferons can play a biological role in antiviral and immunomodulatory, inhibiting specific cell growth and proliferation and killing tumor cells (58, 59). Therefore, interferon therapy has been used to treat common viral diseases such as hepatitis (60) and various cancers (61).

When osteoclasts are induced to produce IFN-β, the binding of IFN-β to its bioreceptor activates ISGF-3 (formed by the aggregation of STAT1, STAT2, and IRF9) *via* the classical JAK/STAT pathway, initiating a signal transduction cascade (62). Then, c-Fos will be inhibited, leading to the inhibition of osteoclast production and activity (11). Thereby, IFN-β forms negative feedback of its own (**Figure 3**). Thus, IFN-β also plays a vital role in regulating bone homeostasis. In addition, osteoclasts express iNOS and release NO, and the NO produced by this pathway also acts as a negative feedback signal to limit RANKL-stimulated osteoclastogenesis (63). In iNOS-deficient bone marrow cells, RANKL-induced NO production was inhibited, leading to an increase in the number of terminally differentiated osteoblasts (64). Direct administration of IFN-β in RAW264.7 cells stimulated iNOS expression in the absence of RANKL, thereby upregulating NO expression. NO, like IFN-β, inhibited osteoclast differentiation. These results suggest that IFN-β may be a key mediator of iNOS-derived NO induction by RANKL in developing osteoclasts and that iNOS can mediate the inhibitory effect of IFN-β on osteoclasts (65).

Another interaction of IFN-β involved in the regulation of bone homeostasis is 4-1BBL with 4-1BB. 4-1BB, also known as CD137, is similar to RANK and is a member of the same TNF receptor family, encoded by the TNFRSF9 gene. Upon its activation, antigen-presenting cells, such as dendritic cells, B cells, and macrophages, express 4-1BBL (66). Osteoclast precursors can express 4-1BB and 4- BBL after exposure to RANKL (67). In BMMs co-stimulated by M-CSF and RANKL, 4-1BBL mRNAs are upregulated (68). In the animal model, 4-1BB knockout mice also showed increased bone mass compared to the wild group. The number of osteoclasts was significantly reduced in the presence of immobilized recombinant 4-1BB (4-1BB-Fc). 4-1BB can induce the binding activity of IRF3, and IRF3 is activated by 4-1BB stimulation, which induces IFN-β (11, 69, 70). It is not difficult to speculate that the decrease in osteoclast activity caused by 4-1BB should be due to the inhibition of c-Fos expression by IFN-β.

### 3.2 Targeting STING/IFN-β in osteoporosis

As a critical signal transduction molecule involved in the innate immune response, STING, triggered by cytoplasmic DNA from pathogens and hosts, can induce type I interferon and pro-inflammatory cytokine secretion, defend against viral and intracellular bacterial infections, and regulate the spontaneous anti-tumor immune response *in vivo*. Targeted STING is a new tool for immunotherapy. In addition to immune or oncological diseases, the role of STING in bone metabolic diseases has been the focus of attention in recent years. DNase II is a nuclease that degrades dsDNA. Lack of DNase II causes DNA accumulation in cells and produces several cytokines, including type I IFN (71). Mice lacking DNase II and IFNAR were able to develop distal aggressive inflammatory arthritis (72). However, this arthritis was eliminated in the absence of STING (71, 73). Surprisingly, the arthritis model (DNase II^-/-^/IFNAR^-/-^ double-knockout mice) showed aberrant accumulation of bone in both long bones and the spleen at sites of local DNA accumulation (13). STING deficiency inhibited bone accumulation (13), revealing a potential role of the STING pathway in bone, although the exact mechanism is unclear.

Patients with advanced cancer often suffer from severe pain due to bone metastases and bone destruction with osteolytic lesions (74). Agonists of the immunomodulator STING have significantly protected against pain (75), bone destruction (13, 19), and local tumor burden (76). One of its effects is alleviating cancerous bone pain by regulating osteoclast function in the tumor microenvironment to prevent local bone destruction, which depends on host-intrinsic STING/IFN-β signaling. Bone metastases in patients with cancer produce osteolytic bone destruction due to tumor-induced osteoclast formation and activation (77). Bone loss was significantly reduced in Lewis lung carcinoma (LLC) or breast cancer mice treated with DMXAA and ADU-S100, two different STING agonists, similar to zoledronic acid (ZA) (18). In contrast, the reversal effect of DMXAA on bone loss was eliminated in the STING knockout group of mice. Thus, the inhibitory effect of STING agonists on bone resorption is dependent on STING. Systemic administration of STING agonists also promotes a robust IFN-I response in the systemic and bone cancer tumor microenvironment. DMXAA treatment does not prevent bone destruction in IFNAR1-deficient mice. IFNAR is required for IFN signaling (57). Thus, the protective effects of STING agonists against cancerous bone destruction require IFN-I signaling (18).

STING, also known as Tmem173, has been shown to inhibit osteoclast differentiation and activity by regulating IFN-β production. It inhibits the expression of osteoclast-specific genes and related enzymes and downregulates the activation of osteoclast-specific transcription factors (20). CDNs are symbiotic bacterial-derived second messengers in the intestine that regulates bacterial survival, colonization, and biofilm formation and have immunomodulatory activity by inducing type I interferon expression by macrophages through the STING signaling pathway (34, 78). CDNs dose-dependently inhibit M-CSF and RANKL-induced differentiation of bone marrow macrophages to osteoclasts and induce phosphorylation of TBK1 and IRF3, representative features of STING activation (19). In contrast, inhibition of osteoclast differentiation was reversed in STING knockdown BMMs. These suggest that the STING signaling pathway plays a crucial role in CDNs-mediated inhibition of osteoclast differentiation. In addition, CDNs increased the expression of IFN-β, a member of the IFN-I family, which has also been identified as a typical negative regulator of RANKL-induced osteoclast differentiation (79, 80). RANKL induces IFN-β expression *via* c-Fos (81). In turn, IFN-β binds to IFNAR on the membrane, which activates ISGF-3 and prevents RANKL-induced c-Fos expression from inhibiting osteoclast differentiation (11). The inhibitory effect of CDNs on osteoclast differentiation was absent in the presence of antibodies blocking IFNAR, and no inhibitory effect was observed in knockout IFNAR macrophages (19). These also confirm that CDNs induce phosphorylation of STAT1, which mediates IFNAR signaling. Experiments performed with a mouse cranial implant model showed that CDNs inhibit RANKL-induced bone resorption (19). These results suggest STING induces IFN-β to inhibit osteoclast differentiation and bone resorption.

It is well known that IFN-I response is a weapon against viruses. IFN-I is induced during STING-mediated immune responses. IFN-I can also be stimulated by the osteoclast-specific gene c-Fos and ultimately inhibits osteoclast production and activation through IFNAR transmission (11, 82). STING regulates the inhibitory effect of IFN-I on osteoclasts, and the knockdown of STING reverses this effect (79). Knockdown of STING can offset this effect (19). Therefore, targeting STING/IFN-β to increase IFN-β expression and thereby inhibit osteoclast bone resorption is expected to be a new approach to treating osteoporosis. In addition, interferon therapy has been used in the clinic. And IFN-β has a relatively good clinical tolerability and safety profile. However, it faces several tests when using IFN-β to treat bone metabolic diseases, including osteoporosis. First, as with other protein drugs, treatment with interferon results in the production of neutralizing antibodies in the patient (83). Second, effective delivery of the drug to the bone microenvironment is another challenge in using IFN-β for treating bone metabolic diseases (84). Targeting STING to increase the level of interferon in the body may solve these problems faced by treatment with interferon alone. In addition, the inhibitory effect of IFN-β on osteoclasts is mainly due to its negative feedback mechanism. However, the action of IFN-β is also inhibited by another kind of negative feedback. Suppressors of cytokine signaling (SOCS)-1 and SOCS-3 in response to RANKL can act as inhibitory factors that significantly inhibit IFN-β signaling (85). Thus IFN-β-mediated inhibition of osteoclastogenesis has a potential counteracting pathway. It may be the same challenge for targeting STING/IFN-β with interferon therapy alone.

## 4 STING/NF-κB and osteoporosis

Past studies have indicated that the cGAS-STING pathway is a key component of the innate immune response as a host defense against multiple pathogens. At the same time, sustained STING activity may lead to fatal inflammatory diseases (39). The continuous secretion of pro-inflammatory cytokines enhances tissue destruction and impairs the organism’s homeostasis, thus affecting functional integrity. NF-κB is a downstream target of STING signaling and can be activated by it. NF-κB is a ubiquitous transcription factor activated by various stimuli, including infection, inflammation, and oxidative stress (86). The aging process is accompanied by a chronic and persistent inflammatory state (87). NF-κB is also a hub of the aging process, promoting transcription and expression of various genes associated with inflammation and can regulate inflammatory signaling during aging induced by oxidative stress (88). NF-κB is associated with many age-related diseases and inflammatory diseases (89), including Alzheimer’s disease (90), diabetes mellitus (91), cancer (92), and autoimmune and inflammatory diseases (93). Activation of NF-κB signaling was found in senescent ARPE-19, and NF-κB was confirmed to be a downstream target of STING in oxidative stress-induced senescent retinal pigment epithelium (RPE) (94). In microgliomas, polyglutamine binding protein 1 (PQBP1) activates cGAS-STING by interacting with sensing extrinsic tau 3R/4R proteins (95). Activation of the PQBP1-cGAS-STING pathway leads to nuclear translocation of NF-κB and expression of inflammatory genes, resulting in brain inflammation and cognitive dysfunction in mice. Psoriasis, a chronic inflammatory skin disease, is associated with innate and adaptive immune responses. STING antagonist H-151 ameliorates psoriasis by inhibiting STING/NF-κB-mediated inflammation (96).

Inflammation is also closely associated with bone metabolism diseases, including osteoporosis (97), osteoarthritis(OA) (98), intervertebral disc degeneration(IVDD) (99), bone lysis (100), and spondyloarthritis (101). STING upregulation was found to be associated with the development of IVDD. And vertebral inflammation mediated by activation of the cGAS/STING molecular pathway is a novel form of animal model used to induce disc degeneration (15). Excessive accumulation of ROS can lead to DNA damage, which activates the cGAS/STING pathway (102). ROS-induced DNA damage is thought to be one of the leading causes of nucleus pulposus (NP) cell degeneration during IVDD progression (103). Moreover, the knockdown of STING expression can attenuate ROS-induced disc degeneration (16). Similarly, pharmacological inhibition of STING also protects NP cells from inflammation-induced apoptosis (17). Moreover, the process of OA is also accompanied by increased expression of STING and NF-κB, and exogenous supplementation with itaconate can inhibit the STING/NF-κB signaling pathway to alleviate the progression of OA (14). Osteoporosis, an age-related disease of bone metabolism, is also an inflammatory disease. Elevated levels of NF-κB can also be found in osteoporosis models. Aging-associated bone loss is characterized by decreased bone formation and increased bone resorption, and it is often referred to as senile osteoporosis (104). Aging is a biological process characterized by changes in the redox state of the organism and inflammatory responses induced by oxidative stress (105). Oxidative stress can release mtDNA (106), which can act as an upstream of the cGAS-STING signaling pathway and activate STING (107). The activation and transduction of STING are crucial in the development and progression of aging-related diseases (108, 109). Therefore the application of targeting STING/NF-κB in osteoporosis is worth exploring.

### 4.1 NF-κB

NF-κB is one of the best-characterized transcription factors that regulate inflammation and innate and adaptive immune responses (110). Activation of NF-κB signaling leads to the production of various inflammatory cytokines, chemokines, adhesion molecules, transcription factors, and antimicrobial effector molecules that initiate and mimic inflammatory responses and coordinate the immediate host response to pathogens and tissue damage. The NF-κB transcription factor family includes five members p50 (NF-κB1), p52 (NF-κB2), RelA (p65), RelB, and c-Rel (111). All NF-κB subunits have a structurally conserved N-terminal sequence spanning 300 amino acid residues called the Rel homology structural domain (RHD) (112). The RHD is responsible for DNA binding, dimerization, and nuclear translocation of NF-κB subunits, which can divide into three structural components - the N-terminal structural domain (NTD), the dimerization structural domain (DD), and the nuclear localization sequence (NLS) polypeptide - all of which mediate the various activities of the RHD and subsequent NF-κB signaling (110, 113, 114). Subunits RelA, RelB, and c-Rel are produced as mature proteins. In contrast, the p50 and p52 subunits are produced by the precursor proteins (113).

In the resting state, NF-κB subunits bind to IκB proteins, inhibiting their activity and maintaining NF-κB subunits in an inactive state (115). In turn, IκB kinase (IKK) can degrade these inhibitory proteins (116). Once activated by upstream signaling cascades, phosphorylated IKK degrades the IκB protein and releases the subunits of the NF-κB. Then these subunits go into the nucleus as dimers and participate in the transcription of various target genes (10). For example, the functional subunits p65 and p50 enter the nucleus and bind to target genes, producing large amounts of inflammatory mediators, and the gene products further activate NF-кB, causing an expanded cascade of uncontrolled inflammatory responses. More and more NF-κB target genes have been identified (117), including various cytokines such as interleukin (IL) and TNF, interferons, and antiapoptotic proteins, such as BIRC2, BIRC3, and BCL2L1.

### 4.2 Relationship between NF-κB and osteoporosis

Bone homeostasis is necessary for the maintenance of normal bone function. Bone homeostasis is maintained by bone remodeling mediated by osteoblasts and osteoclasts, which are responsible for bone formation and resorption (48). Osteoblasts and osteoclasts are the essential cells that regulate bone homeostasis. NF-κB is the master transcription factor that regulates the inflammatory response and bone remodeling process (118, 119). Chronic inflammation induces excess pro-inflammatory cytokines, disrupting homeostasis (120). These result in abnormal bone remodeling, including osteosclerotic and osteolytic lesions (121).

Pro-inflammatory cytokines driven by NF-κB are powerful signals to regulate bone homeostasis (122). Elevated expression of TNF, IL-1, IL-6, and IL-7 has been found in various chronic inflammatory bone diseases, including osteoarthritis (123), osteoporosis (124), and periodontal disease (125). These pro-inflammatory cytokines are all produced by macrophages, lymphocytes, osteoblasts, and bone marrow stromal cells under the regulation of NF-κB and stimulate NF-κB signaling in target cells, which further serves to amplify inflammation (126). Osteoclasts are specialized cells of the monocyte-macrophage lineage responsible for bone resorption. In contrast, osteoblasts are differentiated from mesenchymal stem cells to osteogenesis and are responsible for establishing new bone. NF-κB has an essential role in osteoblasts and osteoclasts, thus affecting bone regulation.

#### 4.2.1 Role of NF-κB in bone resorption

NF-κB signaling is directly involved in the differentiation and activation of osteoclasts responsible for bone resorption (127). The binding of RANKL to RANK triggers a complex and unique signaling cascade that controls lineage commitment and activation of osteoclasts (128). Activating NF-κB signaling in osteoclasts is essential for their differentiation and activation (54). TNF receptor–associated factor (TRAF) proteins are cytoplasmic adaptor proteins that bind to various receptors of the TNF receptor (TNFR) superfamily. An essential role of TRAFs in RANK-RANKL signaling is inducing NF-κB (51). Among TRAFs, TRAF6 is the most critical adaptor of RANK-RANKL-induced osteoclastogenesis (129). Genetic experiments have shown that TRAF6 is required for osteoclast formation and activation (130). Like mice lacking NF-κB p50 and p52 subunits (131), TRAF6-deficient mice develop severe osteoporosis (132).

Usually, NF-κB in the cytoplasm is bound to the inhibitory protein IκB while keeping NF-κB in a resting state. While various stimuli lead to the activation of IKK, which leads to the degradation of IκB bound to the NF-κB subunits, the released NF-κB enters the nucleus as a homodimer or a heterodimer and activates transcription, thus exerting biological effects. For example, released p65 and p50 subunits enter the nucleus for transcription of osteoclast-related genes, thus inducing bone resorption (21). IKK is a complex of three subunits, IKKα (also known as IKK1), IKKβ (also known as IKK2), and IKKγ(also known as NEMO). IKKβ is required for osteolysis *in vitro* and *in vivo*, and the knockdown of IKKβ can lead to bone loss in mice (133). While IKKα is required for RANK ligand-induced osteoclast formation *in vitro*, it is not required *in vivo (* 134). Thus, targeting IKK can regulate the NF-κB activity of osteoclasts and prevent bone loss, providing a new idea for the treatment of osteoporosis (135). In conclusion, NF-κB is an essential mediator of osteoclastogenesis (136), which leads to excessive bone resorption and osteoporosis. Pharmacotherapy can inhibit RANKL-mediated osteoclastic formation by targeting the NF-κB pathway to attenuate inflammatory factors and ROS production and can reduce bone loss *in vivo* in ovariectomized (OVX) model (137).

#### 4.2.2 Role of NF-κB in bone formation

Osteoblasts derived from mesenchymal stem cells (MSCs) are responsible for bone formation. NF-κB activity is suppressed in mature osteoblasts, so NF-κB activation in osteoblasts inhibits bone formation (136). NF-κB activation occurs in bone trabeculae of naturally aging mice (138). Increased NF-κB activity was found in MSCs isolated from aged mice compared to young mice, and inhibition of the NF-κB pathway partially rescued the reduction in osteogenesis in aged MSCs (139). And increased RANKL and reduced OPG expression was observed in aged MSCs, which resulted in increased RANKL/OPG ratio and osteoclast activation. Chronic NF-κB activation has also been shown to impair the differentiation of MSCs along the osteogenic pathway and osteoblast-mediated bone formation (140).

In the absence of NF-κB activation, prolonged c-Jun N-terminal kinase (JNK) activation, which regulates FOSL1 (also known as Fra1) expression, contributes to bone formation (126, 141). Mice specifically lacking IKK-β in osteoblasts exhibit increased bone mass, mainly because reduced NF-κB activity by IKK-β deficiency increases JNK activity and Fra1 expression, ultimately leading to increased bone formation to maintain bone mass in OVX mice (142). Fra1 is an important transcription factor involved in bone matrix formation (143). Chronic inflammation can inhibit bone formation. For example, the pro-inflammatory factor TNF-α inhibits osteoblast differentiation (144), but the IKK inhibitor BAY11-7082 rescues the TNF-α-induced inhibition of osteoblast differentiation by inhibiting NF-κB (145). Thus, inhibition of osteoblast NF-κB can promote bone formation. A decrease in NF-κB activity in osteoblasts leads to an increase in bone formation (146). The NF-κB inhibitor, S1627, upregulates the mRNA of osteoblast-specific genes (such as type I collagen and alkaline phosphatase) to increase osteoblast differentiation and bone formation *in vitro (* 147). Moreover, it can increase bone formation to repair bone defects in a mouse cranial defect model and alleviate osteoporosis in the OVX mouse model. Therefore, targeting NF-κB could provide a novel and effective therapeutic strategy for osteoporosis and other inflammatory bone diseases.

### 4.3 Targeting STING/NF-κB in osteoporosis

Excessive accumulation of ROS leading to redox imbalance and overactive osteoclasts is associated with the progression of osteoporosis. The process of osteoclastogenesis is accompanied by the production of ROS, which plays a role in osteoclast differentiation and bone resorption (52). In addition, ROS-induced mtDNA release induces inflammation through the activation of cGAS/STING (148). ROS can induce NF-κB through the RANKL/RANK cascade reaction, which is further involved in osteoclastogenesis (149). In addition to promoting type I interferon production in the innate immune response (150), STING can also act as an NF-κB upstream of NF-κB, stimulating its production.

In the past, it was generally considered that NF-κB activation *via* STING is exclusively dependent on TBK1. However, studies have now demonstrated that TBK1 is dispensable for NF-κB, although TBK1 and its kinase activity are essential for STING-dependent IRF3 activation and INF-I. In fact, TBK1 and IKK redundantly drive NF-κB activation when the IFN-I reaction is triggered by TBK1 and its homolog, IKK (151). Inhibition by TBK1/IKK kinase indicates that IRF3 activation highly depends on TBK1 kinase activity. In contrast, NF-κB sensitivity to TBK1/IKK kinase inhibition is significantly reduced, and TBK1 was dispensable for NF-κB activation downstream of STING *in vitro* and *in vivo (* 151). So, NF-κB production can be activated by STING through a non-classical pathway, a process that is independent of IFN-β (44). In addition, non-classical STING signaling activates NF-κB pathways mainly through K63-mediated ubiquitination and has no effect on IFN-I (38).

CDNs can inhibit osteoclast differentiation by inducing IFN-β through STING signaling (19), suggesting that activation of STING can inhibit osteoclast differentiation through IFN-β. A sustained activation state of STING can cause a series of inflammatory responses in the organism. NF-κB, acting as a pro-inflammatory gene, is involved in osteoclastogenesis as another downstream of STING. In contrast to NF-κB, nuclear factor erythroid2-related factor 2 (Nrf2), a critical antioxidant molecule, has been shown to inhibit osteoclast formation and bone resorption by reducing ROS (152). In addition, Nrf2 negatively regulates STING signaling (153).

RTA-408 was found to act as an activator of Nrf2 that inhibits STING expression and subsequent NF-κB activation but does not affect IFN-β expression (21). RTA-408 inhibits RANKL-induced K63 ubiquitination of STING by suppressing the interaction between STING and the E3 ubiquitin ligase TRAF6. As a downstream of STING, NF-κB was also inhibited by RTA-408, mainly by suppressing IκBα protein degradation, preventing p65 from translocating to the nucleus and thus rendering NF-κB inactive. Overexpression of STING rescued the inhibitory effect of RTA-408 on NF-κB signaling and osteoclastogenesis. *In vivo* experiments showed that RTA-408 attenuated osteoclastogenesis-induced bone loss in C57BL/6 mice by inhibiting STING-mediated NF-κB (21). Thus, inhibition of STING-dependent NF-κB signaling could inhibit osteoclastogenesis and reduce bone loss. Targeting STING/NF-κB may be a promising pathway for the future treatment of osteoporosis.

## 5 STING/type H vessels and osteoporosis

In recent years, it has been found that in addition to osteogenic and osteoclastic effects, angiogenesis also plays a vital role in bone homeostasis in the mammalian skeletal system. Type H vessels that can induce bone formation have been discovered recently and are named for their high expression of EMCN and CD31 (154). Angiogenesis, the development of new blood vessels from pre-existing vessels, is closely associated with osteogenesis during skeletal development and bone remodeling. Blood vessels provide bone tissue with essential nutrients, oxygen, growth factors, and hormones and play a crucial role in the regulation of bone formation (155).

### 5.1 Relationship between type H vessels and osteoporosis

Osteogenesis is linked to angiogenesis (156). The close spatial and temporal link between osteogenesis and angiogenesis has been termed “angiogenesis-osteogenesis coupling” (157). Type H vessels are located near the epiphyseal growth plate, the epiphyseal periosteum, and the endosteum. Type H vessels are densely surrounded by osteoprogenitors expressing Osterix, a potent promoter of bone formation (158). These osteoprogenitor cells can differentiate into osteoblasts and osteocytes. Under aging conditions, osteoblasts are significantly reduced in the long bones of mice (159), which is associated with a decrease in Type H vessels and reduced bone mass (160). The abundance of Type H vessels is an essential indicator of bone loss in elderly subjects and patients with osteoporosis (161).

PDGF-BB is a chemotactic and mitogenic factor of the PDGF family, produced by hematopoietic stem cells (162). It is essential for promoting the migration, proliferation, and differentiation of various mesenchymal cell types, such as endothelial progenitor cells and mesenchymal stem cells, to promote angiogenesis and osteogenesis (163, 164). PDGF-BB enhanced type H vessels and bone formation during bone plastination and remodeling. The concentration of PDGF-BB was decreased in the OVX mouse model (165). Pharmacological stimulation was able to secrete PDGF-BB to stimulate H-type angiogenesis, thereby promoting osteogenesis to prevent bone loss in OVX mice (166). Glucocorticoids reduce vascularity and blood flow to the bone, causing osteonecrosis and bone loss (167, 168). Glucocorticoid-induced osteoporosis (GIO) is also common osteoporosis. In GIO mouse models, glucocorticoids inhibit PDGF-BB secretion by pre-osteoblasts, inhibiting Type H vessels and reducing osteogenic capacity (169). And L-235, a cathepsin K inhibitor, prevents bone loss by inhibiting osteoclast-inducing bone resorption while maintaining PDGF-BB secreted by preosteoclasts preserving Type H vessels (169).

HIF-1α is a transcription factor that mediates the cellular response to an altered oxygen environment and controls angiogenesis (170).HIF-1α plays a crucial role in bone formation, regeneration plays a key role in bone formation and regeneration, and its expression and activity are regulated by hypoxia Oxygen is required for the high metabolic demand of osteoblasts. Therefore, osteoblasts and nearby ECs may increase HIF-1α expression during relative hypoxia during osteogenesis (155). And ECs express HIF-1α at high levels in young mice, which decreases with age and is associated with a decrease in ECs and age-dependent bone loss. Activation of hypoxic signaling in ECs increased the number of Type H vessels and enhanced angiogenesis and osteogenesis (171). EC-specific deletion of HIF-1α resulted in a significant decrease in osteoblast producers and was associated with reduced trabecular formation. Thus, HIF-1α signaling is vital in regulating type H vascular abundance and couples angiogenesis to osteogenesis (172). Tetramethylpyrazine activates the AMPK/mTOR/HIF-1α signaling pathway to induce type H vessel angiogenesis and improve bone homeostasis in aging mice (173). This provides an additional therapeutic target for the treatment of age-related osteoporosis.

### 5.2 Targeting STING/type H vessels in osteoporosis

Type H vessels also play an indelible role in bone remodeling. Osteoblasts, osteoclasts, and periosteal cells interact with vascular endothelial cells. The STING signaling pathway may act directly on these cells, thereby affecting the angiogenic process. Several studies have shown an association between STING and angiogenesis. STING is expressed in endothelial cells, and cGAMP leads to the activation of cGAS-STING in endothelial cells (174, 175). STING-associated vascular disease (SAVI), with onset in infancy, is an autoinflammatory disease caused by mutations in STING function, which can cause vascular and pulmonary syndromes and cause systemic inflammatory responses (176, 177).

Previous studies have shown that STING can affect angiogenesis in multiple ways. In the zebrafish xenograft model, exogenous administration of cGAMP can activate STING-dependent STAT3, leading to the inhibition of tumor vascular proliferation and migration (178). Palmitic acid (PA) induces mtDNA into the cytoplasm by inducing mitochondrial damage, activating the cGAS-STING-IRF3 signaling (179). Activation of the cGAS-STING-IRF3 pathway dysregulates the Hippo-YAP pathway and inhibits angiogenesis. In addition, STING-IRF3 can trigger endothelial inflammation in response to PA-induced mitochondrial damage (180). In retinal microangiopathy, mtDNA drives inflammation of microvascular endothelial cells *via* the cGAS-STING signaling pathway (181). Inflammation in the physiological state is a protective mechanism for tissue damage and the basis for tissue repair and regeneration. Nevertheless, an excessive inflammatory response can impair the integrity of the tissue and its function. The persistent inflammatory state of the vascular endothelium leads to impaired angiogenesis and poor bone healing, which affects bone reconstruction (182).

Activation of the STING signaling pathway impairs angiogenesis, including type H vessels. In addition, the prolonged inflammatory response stimulated by the STING pathway delays the bone healing process. Activation of STING inhibits angiogenesis both *in vitro* and *in vivo* and slows the bone healing process *in vivo (* 12). Conversely, inhibition of STING accelerated bone healing by enhancing type H vessel formation during coupled osteogenesis (12). Therefore, targeting STING to enhance type H vessel formation and thus promote osteogenesis provides a new idea for the treatment of osteoporosis.

## 6 Conclusions

Osteoporosis is a bone metabolic disease and an aseptic inflammatory disease. In recent years, bone immunology has become a hot research topic in bone metabolic diseases by studying the functional interactions between the skeletal and immune systems, including various cytokines and transcription factors that affect both systems, to explore new immunological therapeutic avenues for bone metabolic diseases. STING is the core of natural immunity and a new target for immunotherapy. The core of IFN-β treatment for osteoporosis is also inseparable from bone immunology. With the discovery that IFN-β can play a unique role in regulating bone homeostasis, targeting the STING/IFN-β signaling pathway is also emerging as a potential therapeutic tool for osteoporosis. However, the STING pathway has a dual role in bone metabolism. In addition to its immune function, STING can also act as an inflammatory protein to induce NF-κB, thereby mediating the development and progression of various inflammatory diseases, including osteoporosis. But in studies on osteoclastic inhibition by STING/IFN-β, the effect of another STING downstream signaling molecule, NF-κB, had rarely been considered, which is a drawback of related studies. If NF-κB is not disturbed in the activation of STING/IFN-β pathway to inhibit osteoclastic resorption, it will be a more rigorous and appropriate choice. The classical STING/NF-κB pathway, the cGAS-STING pathway, is dependent on the activation of TBK1, which not only activates NF-κB but also mediates the production of IFN-β by activating IRF3. In contrast, the non-classical STING/NF-κB pathway blocks the production of IRF3/IFN-β in parallel. Inhibition of NF-κB activation and, thus, osteoclast differentiation by targeting STING without affecting the level of IFN-β has been shown to alleviate bone loss in the OVX mice. Therefore, targeting the STING/NF-κB pathway is also expected to be a new therapy for osteoporosis. In addition, activation of STING leads to a prolonged inflammatory response that delays revascularization and inhibits the production of type H vessels, which are closely related to osteogenesis and can induce bone formation. Targeting STING/type H vessels also promotes bone reconstruction and osteogenesis by promoting tape H vessel formation. To enhance bone formation and inhibit bone resorption, a STING inhibitor would be a reasonable choice if it were designed to specifically target NF-κB rather than IFN-β.

Overall, STING has a unique role in osteoporosis. The drugs commonly used in clinical to treat osteoporosis are mainly bisphosphonates, which inhibit bone resorption, and calcitonin and estrogen drugs, which can also promote osteoblastogenesis, but they all have many side effects. Therefore, how to safely and effectively treat osteoporosis remains a challenge to tackle. Targeted STING has been applied in antiviral immunotherapy and does represent a rather promising therapeutic option in the new field of treatment of osteoporosis. Targeted STING not only has the ability to directly inhibit osteoclast-mediated bone resorption and promote osteoblast-mediated bone formation but also can promote type H vascular angiogenesis to indirectly enhance the osteogenic effect. However, the network of regulatory pathways involved in STING is also very complex, and the decrease in IFN-β production-mediated bone resorption due to STING activation is in contradiction with the increase in bone resorption and decrease in bone formation mediated by NF-κB activation and type H vascular inhibition. Targeting STING as a therapeutic option for osteoporosis requires balancing these conflicting biological effects. Therefore, there is still much room for exploration of the STING pathway in bone metabolism.

## Author contributions

ZG (1^st^ author) conceived and drafted the manuscript. HZ, SH, YZ, and XL discussed the concepts of the manuscript. ZG (1^st^ author) and ZG (2^nd^ author) drew the figures. YZ and XL reviewed and revised the manuscript. All authors contributed to the article and approved the submitted version.
